# Changes in serum angiogenic factors among patients with acute pain and subacute pain

**DOI:** 10.3389/fnmol.2022.960460

**Published:** 2022-07-15

**Authors:** Xuewei Yang, Chunmei Yuan, Huanling Wang, Yunxia Wang, Mei Liu, Zongjin Li, Jun Zhang

**Affiliations:** ^1^Department of Anesthesiology and Pain Medical Center, Tianjin Union Medical Center, Nankai University, Tianjin, China; ^2^School of Medicine, Nankai University, Tianjin, China

**Keywords:** angiogenic factors, acute pain, postherpetic neuralgia, low back pain, trigeminal neuralgia, TSP-2

## Abstract

Screening serum biomarkers for acute and subacute pain is important for precise pain management. This study aimed to examine serum levels of angiogenic factors in patients with acute and subacute pain as potential biomarkers. Serum samples were collected from 12 healthy controls, 20 patients with postherpetic neuralgia (PHN), 4 with low back pain (LBP), and 1 with trigeminal neuralgia (TN). Pain intensity in these patients was evaluated using the visual analog scale (VAS). The serum concentrations of 11 angiogenic biomarkers were examined by Milliplex Map Human Angiogenesis Magnetic Bead Panel 2. The pain assessment from VAS showed that all patients showed moderate and severe pain. Among 11 angiogenic factors, osteopontin (OPN), thrombospondin-2 (TSP-2), soluble platelet endothelial cell adhesion molecule-1 (sPECAM-1), soluble urokinase-type plasminogen activator receptor (suPAR), and soluble epidermal growth factor receptors (sErbB2) were up-regulated and soluble interleukin-6 receptor α (sIL-6Rα) were down-regulated in patients with pain compared to the healthy participants (all *P*-values were < 0.005). Moreover, a linear regression model showed that the serum OPN concentration was correlated with pain intensity in patients with PHN (*P* = 0.03). There was no significant difference between the serum concentration of soluble epidermal growth factor receptors, sErbB3, soluble AXL, tenascin, and soluble neuropilin-1 in patients with acute and subacute pain and that of healthy controls. The results of this study provided new valuable insights into our understanding of angiogenic factors that may contribute to as mechanistic biomarkers of pain, and reveal the pathophysiological mechanism of pain.

**Clinical Trial Registration:** www.chictr.org.cn, identifier ChiCTR2200061775.

## Introduction

Pain may be conceived as a disease in conditions, such as fibromyalgia or non-specific low back pain (LBP) (Reckziegel et al., 2019; Treede et al., 2019). More popularly, pain is a leading complaint in some diseases such as postherpetic neuralgia (PHN), trigeminal neuralgia (TN), peripheral nerve injury, painful polyneuropathy, etc. (Scholz et al., 2019; Treede et al., 2019). The cost of acute or subacute pain due to direct medical treatment and productivity lost represents a heavy economic burden. Moreover, therapeutic management of pain is challenging, and complete relief is uncommon, owing to efficacy limitations of current treatments (Scholz et al., 2019). It is also intricately related to opiate addiction and has a significant impact on disability and overdose deaths. To combat these massive societal burdens, more effort is required to develop novel tools and therapies for combating chronic pain and reducing reliance on opiates for managing chronic pain (Reckziegel et al., 2019). This effort has emphasized the urgent need to develop validated biomarkers, with the assumption that such biomarkers would, in turn, facilitate mechanically driven development of novel therapies (Reckziegel et al., 2019). The current study aim to identify new biomarkers for acute and subacute pain by examining blood samples of patients with pain, and to identify clues for pain pathogenesis.

Angiogenesis, which is the formation of new blood vessels from the endothelium of the existing vasculature, is a fundamental process that occurs in physiological and pathophysiological conditions (Sajib et al., 2018). Insufficient or excessive blood vessel growth underlies many diseases, including cardiovascular and cerebrovascular diseases, osteoarthritis and cancer (Lawton et al., 2015; Viallard and Larrivée, 2017; Sajib et al., 2018). Normal angiogenesis and angiogenic signaling in pathological conditions are mediated by soluble growth factors, membrane-bound receptors, and cell-cell and cell-matrix interactions (Sajib et al., 2018). These factors may also play a role in pain pathogenesis by enhancing inflammation and inappropriate sensory innervation of local tissues (Mapp and Walsh, 2012). Angiogenesis-related signaling factors such as interleukin-1β (IL-1β), IL-6, tumor necrosis factor-α, and cyclooxygenase 2 are known to contribute to pain hypersensitivity by inducing the production of prostaglandins and other proalgesic agents, which activate nociceptors (Mafu et al., 2018). Moreover, previous studies have shown that angiogenesis contributes to pain in osteoarthritis patients and shoulder pain of breast cancer survivors (Mapp and Walsh, 2012; Mafu et al., 2018). All of the above studies shed light on the role of angiogenesis in pain pathogenesis.

We examined the serum levels of 11 angiogenic factors in patients with PHN, LBP, or TN using the Millipore Milliplex Map Human Angiogenesis Magnetic Bead Panel 2 assay and evaluated the correlations of these factors with pain intensity in PHN patients. These 11 angiogenic factors included osteopontin (OPN), thrombospondin-2 (TSP-2), soluble platelet endothelial cell adhesion molecule-1 (sPECAM-1), soluble urokinase-type plasminogen activator receptor (suPAR), soluble epidermal growth factor receptors (sEGFR/sHER1/sErbB1), soluble human epidermal growth factor receptor 2 (sEGFR2/sErbB2s/HER2), sErbB3 (sEGFR3/sHER3), soluble interleukin-6 receptor α (sIL-6Rα), soluble AXL (sAXL), tenascin C (TN-C), and soluble neuropilin-1 (sNRP-1). We will observe the expression levels of their homologous genes in the dorsal root ganglion (DRG), spinal cord, and the key parts of the sensory nervous system using a mouse neuropathic pain model in the next step.

## Methods

### Patients

All the participants were recruited from the Tianjin Union Medical Centre, Tianjin, China. There were 20 patients with PHN, 4 with LBP, and 1 with TN. All the patients were diagnosed by clinically qualified doctors. Twelve age-matched healthy participants without pain-related diseases or conditions were enrolled from the physical examination center of the Tianjin Union Medical Centre. Baseline characteristics (age and sex) of the participants are listed in Table 1. All the patients were notified of no analgesic medication 24 h before the study visit. Ethical approval for this research was approved by the ethical committee of Tianjin Union Medical Centre (approval number: 2016-B08).

**TABLE 1:** Baseline characteristics of healthy controls and patients with PHN, LBP, or TN.

	HC	PHN	LBP	TN
Total, n	12	20	4	1
Gender (M/F)	6M/6F	11M/9F	3M/1F	1F
Mean age in years, range	66.7 (50–82)	74.8 (60–85)	76.5 (73–81)	64

HC, healthy controls; PHN, postherpetic neuralgia; LBP, low back pain; TN, trigeminal neuralgia; M, male; F, female.

### Pain assessment

All the patients and healthy participants completed a visual analog scale (VAS) questionnaire for pain. The participants were asked to rate their current pain intensity on a VAS, ranging between “0–10.” A VAS of “0” represents “no pain”; “1–3” represents mild tolerable pain; “4–6” represents moderate tolerable pain and sleep was disturbed. A VAS pain score from of “7–10” represents intense intolerable pain and both sleep and diet were disturbed.

### Serum preparation

Blood samples from the participants were collected into 10 mL sterile tubes containing no additives. The samples were immediately centrifuged at 1,000 × g for 10 min and sediment-free serum samples were obtained, aliquoted, and frozen at –80°C until further analysis.

### Milliplex map assay for angiogenic factors

Serum concentrations of the 11 angiogenic factors (OPN, TSP-2, sPECAM-1, suPAR, sHER2, sIL-6Rα, sEGFR, sHER3, sAXL, TN-C, and sNRP-1) were measured by commercially available Milliplex Map Human Angiogenesis Magnetic Bead Panel 2 assay (EMD Millipore, Darmstadt, Germany). The serum samples were diluted to 1:5 before use. All procedures were carried out according to manufacturer’s the instructions and all the samples were tested along with the quality controls and standard samples provided in the kit. After all steps were completed, the 96-well plate was placed onto Luminex^®^ 200™ and analyzed using the Bio-Plex Manager software. The equipment settings were provided in the instruction book. Results were expressed in nanogram per milliliter (ng/mL) for the serum levels.

### Statistical analysis

Serum levels of the angiogenic factors were evaluated using absolute concentration values. Data are reported as mean ± standard error of the mean (SEM). Comparisons of each angiogenic factor between the patients with pain and healthy controls was performed using the Student’s *t*-test.

Correlations between the concentration of the angiogenic factors and pain intensity or duration in patients with PHN were determined using linear regression analysis. The F-test was used to determine statistical significance, with *P* < 0.05 considered as significant. Spearman’s order correlation was performed to examine the correlation and calculate the correlation coefficients.

All analyses were performed using Statistical Package for the Social Sciences (SPSS; Chicago, IL, United States). Statistical significance was set at *P* < 0.05.

## Results

### Descriptive characteristics

**FIGURE 1:**
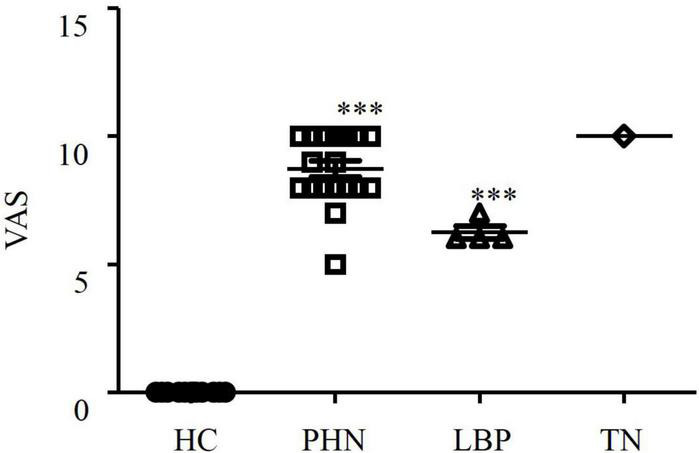
Comparison of visual analog scale (VAS) in healthy controls and patients with postherpetic neuralgia (PHN), low back pain (LBP), or trigeminal neuralgia (TN). ^***^P < 0.001.

Descriptive characteristics of the patients are shown in Table 1. Male and female patients were enrolled randomly in each group (Table 1). All the patients were aged > 55 years. Pain duration varied among the patients (Table 2). According to the classification of acute, subacute and chronic pain, most patients with PHN were classified as having acute pain with a duration less than 3 months. There was only one patient with subacute pain and two with chronic pain in the PHN patients. There was one LBP patient with acute pain and three LBP patients with subacute pain. One patient with TN was classified as having subacute pain. All the patients experienced medium to severe pain (Figure 1). The mean VAS was 8.7 in the PHN patients and 6.3 in the LBP patients, respectively. The mean VAS score of the TN patient was 10.

**TABLE 2:** Classification of patients with pain as acute, subacute, and chronic pain.

		Numbers
	Acute pain (0–3 m)	15
PHN	Subacute pain (3–6 m)	2
	Chronic pain (>6 m)	2
	Acute pain (0–3 m)	1
LBP	Subacute pain (3–6 m)	3
	Chronic pain (>6 m)	0
	Acute pain (0–3 m)	0
TN	Subacute pain (3–6 m)	1
	Chronic pain (>6 m)	0

PHN, postherpetic neuralgia; LBP, low back pain; TN, trigeminal neuralgia.

### Levels of serum angiogenic factors in the postherpetic neuralgia and low back pain patients

**FIGURE 2:**
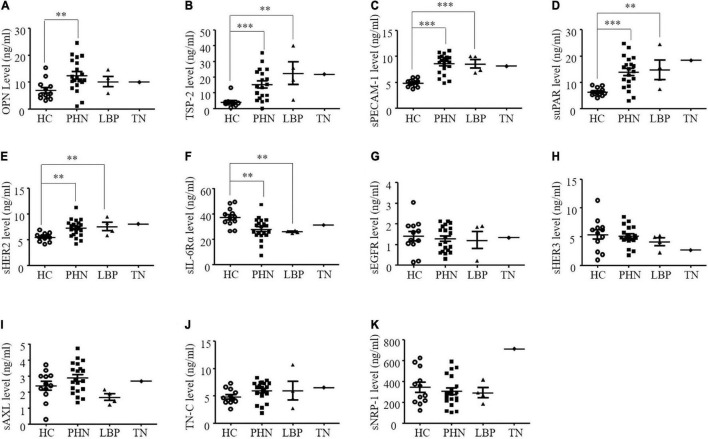
**(A-K)** Comparison of serum angiogenic factors in healthy controls (HC) and patients with postherpetic neuralgia (PHN), low back pain (LBP), or trigeminal neuralgia (TN). **P < 0.01; ^***^P < 0.001. OPN, osteopontin; TSP-2, thrombospondin-2; sPECAM-1, soluble platelet endothelial cell adhesion molecule-1; suPAR, soluble urokinase-type plasminogen activator receptor; sErbB2, soluble human epidermal growth factor receptor 2; sIL-6Rα, soluble interleukin-6 receptor α; sEGFR, soluble epidermal growth factor receptors; sErbB3, soluble human epidermal; sAXL, soluble AXL; TN-C, tenascin C; sNRP-1, solubleneuropilin-1.

The mean ± S.E.M. levels of the serum angiogenic factors are shown in Table 3. Serum OPN, TSP-2, sPECAM-1, suPAR, and sErbB2 levels were significantly higher in patients with PHN (Figures 2A–E), while the serum sIL-6Rα levels were lower in the PHN patients than in the healthy controls (Figure 2F). Similarly, in patients with LBP, serum levels of TSP-2, sPECAM-1, sHER2, and suPAR were significantly increased, while serum sIL-6Rα levels were significantly decreased compared to those in the healthy control group (Figures 2B–F). However, the serum OPN level in patients with LBP didn’t differ from those in the healthy controls (Figure 2A). The serum concentrations of OPN, TSP-2, sPECAM-1, sErbB2, and suPAR in TN patients were higher than those in the healthy controls (Figures 2A–E), while serum sIL-6Rα in the TN patient was lower than the mean value of the healthy controls (Figure 2F). The other five angiogenic factors, including sEGFR, sErbB3, sAXL, TN-C, and sNRP-1 didn’t show any significant differences in patients with pain compared to the healthy controls (Figures 2G–K).

**TABLE 3:** Serum levels of angiogenic factors in healthy controls and patients with PHN, LBP, or TN.

	HC (*N* = 12)	PHN (*N* = 20)		LBP (*N* = 4)		TN (*N* = 1)
	$\bar{\text{X}}$ ± SEM	$\bar{\text{X}}$ ± SEM	*P*	$\bar{\text{X}}$ ± SEM	*P*	X
OPN (ng/mL)	6.94 ± 1.02	12.45 ± 1.31	0.007**	10.14 ± 1.63	0.16	1.00
TSP-2 (ng/mL)	4.37 ± 0.94	16.02 ± 2.02	0.0007***	22.32 ± 6.24	0.002**	21.82
sPECAM-1 (ng/mL)	4.85 ± 0.20	8.54 ± 0.41	0.0000***	8.52 ± 0.73	0.0000***	8.12
suPAR (ng/mL)	6.22 ± 0.44	13.81 ± 1.31	0.0002***	14.62 ± 3.19	0.001**	18.33
sHer2 (ng/mL)	5.48 ± 0.22	7.25 ± 0.36	0.002**	7.54 ± 0.69	0.004**	8.04
sIL-6Ra (ng/mL)	37.2 ± 2.03	27.73 ± 1.91	0.004**	25.74 ± 0.42	0.009**	31.20
sEGFR (ng/mL)	1.65 ± 0.18	1.28 ± 0.13	0.13	1.21 ± 0.35	0.28	1.34
sHer3 (ng/mL)	5.34 ± 0.78	5.09 ± 0.36	0.76	4.09 ± 0.58	0.42	2.65
sAxl (ng/mL)	2.38 ± 0.26	2.87 ± 0.21	0.16	1.67 ± 0.18	0.17	2.68
TN-C (ng/mL)	4.81 ± 0.37	5.92 ± 0.41	0.08	5.94 ± 1.47	0.34	6.54
sNRP-1 (ng/mL)	343.25 ± 46.38	304.39 ± 31.54	0.49	290.53 ± 41.74	0.57	713.12

**P < 0.01; ***P < 0.001.

HC, healthy controls; PHN, postherpetic neuralgia; LBP, low back pain; TN, trigeminal neuralgia; OPN, osteopontin; TSP-2, thrombospondin-2; sPECAM-1, soluble platelet endothelial cell adhesion molecule-1; suPAR, soluble urokinase-type plasminogen activator receptor; sHER2, soluble human epidermal growth factor receptor 2; sIL-6Rα, soluble interleukin-6 receptor α; sEGFR, soluble epidermal growth factor receptors; sHER3, soluble human epidermal growth factor receptor 3; sAXL, soluble AXL; TN-C, tenascin C; sNRP-1, soluble neuropilin-1.

### Associations between angiogenic factors and visual analog scale in the postherpetic neuralgia patients

**FIGURE 3:**
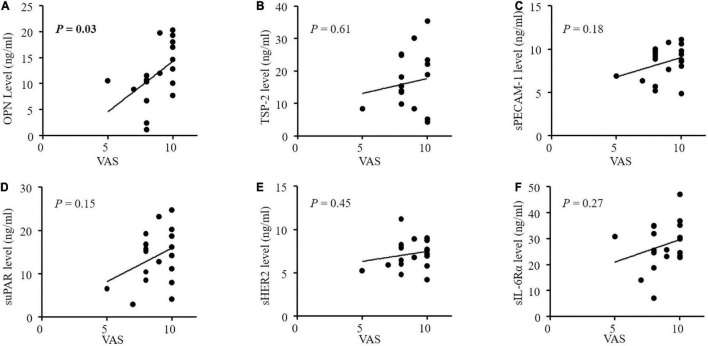
**(A-F)** Correlations between concentrations of angiogenic factors and pain intensity by VAS in patients with postherpetic neuralgia (PHN). Only serum OPN levels were significantly correlated with VAS in PHN patients (*P* = 0.03). OPN, osteopontin; TSP-2, thrombospondin-2; sPECAM-1, soluble platelet endothelial cell adhesion molecule-1; suPAR, soluble urokinase-type plasminogen activator receptor; s ErbB2, soluble human epidermal growth factor receptor 2; sIL-6Rα, soluble interleukin-6 receptor α; VAS, visual analog scale.

**FIGURE 4:**
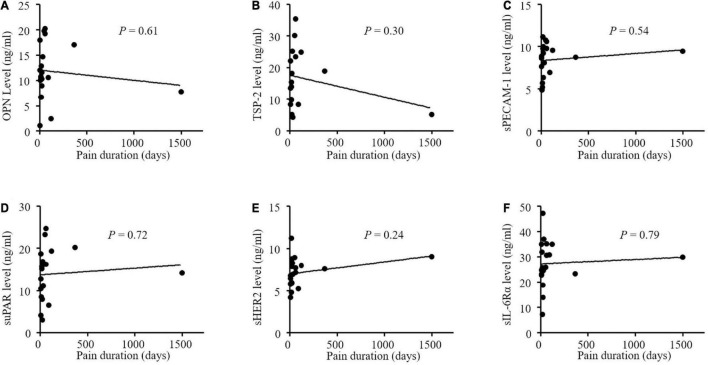
**(A-F)** No correlations between concentrations of angiogenic factors and pain duration in patients with postherpetic neuralgia (PHN). OPN indicates osteopontin; TSP-2, thrombospondin-2; sPECAM-1, soluble platelet endothelial cell adhesion molecule-1; suPAR, soluble urokinase-type plasminogen activator receptor; s ErbB2, soluble human epidermal growth factor receptor 2; sIL-6Rα, soluble interleukin-6 receptor α; VAS, visual analog scale.

The altered angiogenic factors including OPN, TSP-2, sPECAM-1, sHER2, suPAR, and sIL-6Rα in the PHN patients were further analyzed according to pain intensity and duration (Figure 3). We found that only serum OPN level was significantly correlated with VAS scores in the patients with PHN (*P* = 0.03, Figure 3A). The correlation coefficient between serum OPN level and VAS scores in patients with PHN was 0.49. Other factors were not significantly correlated with VAS in the PHN patients although higher levels of TSP-2, sPECAM-1 sErbB2, suPAR and a lower level of sIL-6Rα were found in the serum of PHN patients (Figures 3B–F). None of these angiogenic factors were significantly correlated with pain duration (Figures 4A–F).

## Discussion

The objective of this study was to compare the levels of angiogenic factors in the sera of patients with pain and healthy controls. We found that higher levels of OPN, TSP-2, sPECAM-1, sErbB2, and suPAR and lower levels of sIL-6Rα were up-regulated and sIL-6Rα were down-regulated in those patients with pain compared to the healthy participants. Moreover, serum OPN levels were correlated with pain intensity in patients with PHN. These angiogenic factors may be involved in the pathogenesis of pain, and their soluble part in the blood can serve as biochemical indicators of acute or subacute pain.

### Function of osteopontin in health and disease

The biological functions of OPN are diverse as it can be beneficial for wound healing, bone homeostasis, and extracellular matrix function, and deleterious in cardiovascular diseases, cancer, diabetes, and kidney stone diseases (Icer and Gezmen-Karadag, 2018; Lamort et al., 2019). Circulating OPN has also been indicated in some pain conditions as elevated plasma OPN levels have been found in patients with pain and unstable angina or multiple sclerosis (Soejima et al., 2006; Agah et al., 2018). A statistically significant correlation between OPN in synovial fluid and pain has been found in symptomatic patients with primary knee osteoarthritis and in patients who underwent anterior cruciate ligament reconstruction surgery (Yamaga et al., 2012; Calvet et al., 2018). Osteopontin has been reported to upregulate the expression of IL-6 and IL-8 cytokines in chondrocytes isolated from human osteoarthritis knee cartilage (Yang et al., 2014). Moreover, OPN in the peripheral DRG may contribute to the mechanisms of neuropathic pain. In the DRG, 25% of neurons were immunoreactive for OPN. These neurons were mostly large and exhibited parvalbumin-immunoreactivity, but not calcitonin gene-related peptide immunoreactivity (Ichikawa et al., 2000). Nerve injury induced an increase in OPN in DRG sensory neurons, and OPN knockout reversed mechanical hypersensitivity in mice with neuropathic pain (Ichikawa et al., 2000; Marsh et al., 2007). In the present study, we found an increased OPN levels in the sera of patients with PHN, which was positively associated with pain intensity in the PHN patients. This strongly supports that OPN exerts a role in pain pathogenesis, which may serve as a potential target for pain management.

### Function of PECAM-1 in health and disease

Platelet endothelial cell adhesion molecule-1, also called CD31, is a 130-kDa protein belonging to the Ig superfamily; it has distinct variants and therefore different isoforms which allow diversity of PECAM-1 functions, including inflammation, angiogenesis and vascular development (Kalinowska and Losy, 2006). Splicing of exon 9, which encodes the transmembrane domain, results in the formation sPECAM-1, a soluble form of PECAM-1 (Goldberger et al., 1994) may result in PECAM-1 inhibition (Kalinowska and Losy, 2006). Thus, PECAM-1 is also involved in pain. For example, the sevenfold higher density of PECAM-1-positive capillaries in the cell body-rich area of mouse DRG than in the cell fiber-rich area of the DRG or the sciatic nerve facilitated many potentially neurotoxic agents by preferentially accumulating and injuring cells within the DRG, thereby inducing peripheral sensory neuropathy (Jimenez-Andrade et al., 2008). Moreover, PECAM-1 may also contribute to spinal mechanisms of pain because peripheral nerve injury induces an increase in PECAM-1 immunoreactivity in related spinal cord (Rutkowski et al., 2002; Sweitzer et al., 2002). In the blood circulation, PECAM-1 may mediate the analgesic effect of endomorphin on inflammatory pain by recruiting immunocytes containing β-endorphin to sites of painful inflammation (Sweitzer et al., 2002). The above studies have suggested various roles of PECAM-1 in multilevel pain transmission and various pain conditions. Herein, we found an increase in serum sPECAM-1 levels in patients with pain. Soluble PECAM-1, which may originate from endothelial cells, along with PECAM-1, may result in altered immune cell-endothelial cell interactions and thus, affect pain behaviors under pain conditions (Goldberger et al., 1994; Onore et al., 2012).

### Function of ErbB2 in health and disease

ErbB2 is a member of the EGFR family of receptor tyrosine kinases, which comprises ErbB1/EGFR/HER1, ErbB2/HER2, ErbB3/HER3, and ErbB4/HER4 (Moasser, 2007; Ho et al., 2017). ErbB2 is thought to be an oncogene and has become a target for a number of targeted anti-cancer drugs (Moasser, 2007; Al-Dasooqi et al., 2009). Soluble ErbB2, generated from ErbB2 proteolytic cleavage, includes only the extracellular domain of ErbB2 (Perrier et al., 2018). Soluble HER2 has been detected in the blood of cancer patients, and anticancer strategies targeting it are being developed (Dokala and Thakur, 2017; Perrier et al., 2018). In the present study, we found an increase in sErbB2 but not in sErbB1 or sErbB3 in patients with pain. Clinical and experimental studies have shown that EGFR/ErbB1 inhibitors can relieve neuropathic and breast cancer-related pain (Kaufman et al., 2010; Kersten and Cameron, 2012; Kersten et al., 2013, 2015, 2019; Martin et al., 2017; Wang et al., 2019). Moreover, EGFR/ErbB1 has been implicated in peripheral mechanisms of neuropathic pain (Martin et al., 2017; Wang et al., 2019). Therefore, ErbB2 signaling may be involved in the pathogenesis of pain. However, further studies are required to support this hypothesis.

### Function of IL-6 in health and disease

Interleukin-6 is a key cytokine in many inflammatory and autoimmune diseases and IL-6, as well as IL-6 signaling, have been implicated in pain pathogenesis (Kawasaki et al., 2008; Assier et al., 2010; Boettger et al., 2010; Rendina et al., 2018). The IL-6 receptor is a heterodimer composed of an α chain (IL-6Rα) and a transmembrane chain gp130. Interleukin-6 can also induce cell signaling via sIL-6Rα which is generated by proteolysis of the transmembrane form of IL-6Rα, or by alternative splicing of the messenger RNAs for the α chain. Soluble IL-6Rα allows cells that do not express IL-6Rα to respond to IL-6 (Assier et al., 2010). Increased sIL-6R levels have been found in the sera of Paget disease patients with LBP, suggesting an enhanced transmission of IL-6 signaling in the specialized neural system linked to irregular perception in these patients (Rendina et al., 2018). In DRGs, IL-6 and IL-6R mRNA are expressed in DRG neurons and satellite glial cells, and are significantly elevated in response to peripheral nerve injury (Brázda et al., 2013). Moreover, IL-6, as well as sIL-6Rα, induced long-lasting robust sensitization of joint nociceptors to mechanical stimuli in an experimental arthritis model (Boettger et al., 2010). Spinal injection of sIL-6Rα produces heat hyperalgesia and sIL-6Rα and IL-6 enhance spinal central sensitization (Kawasaki et al., 2008). All these studies provide strong evidence of the close correlation between IL-6 signaling and pathological pain development. Currently, we observed decreased levels of sIL-6Rα in the sera of patients with pain. As a high level of sIL-6Rα is indicative of inflammation and increased nociception, sIL-6Rα levels in these patients with acute or subacute pain conflicts with our expectation. A study on a monoclonal antibody (tocilizumab) to transmembrane and sIL-6Rα showed that tocilizumab was effective in patients with rheumatoid arthritis although tocilizumab increased serum levels of IL-6 and sIL-6Rα. It has been suggested that sIL-6Rα is taken up by immune complexes which might increase the half-life of IL-6Rα and sIL-6Rα (Nishimoto et al., 2008). Therefore, an unidentified complex may be responsible for the decrease in sIL-6Rα levels in the blood of patients with pain observed in the current study.

### Function of uPAR in health and disease

Urokinase-type plasminogen activator receptor is a glycosyl-phosphatidylinositol-anchored membrane glycoprotein belonging to the plasminogen activator system (Irigoyen et al., 1999; Thunø et al., 2009). Soluble uPAR, a free soluble receptor, is cleaved from uPAR. Soluble uPAR levels can be determined in the blood and have been used as biomarkers for chronic inflammatory conditions (Thunø et al., 2009). Elevated serum suPAR levels have been found in patients with chest pain osteoarthritis and migraine with attacks and aura (Tang et al., 2010; Lyngbæk et al., 2013; Yılmaz et al., 2017). Similarly, we postulate that suPAR and its related plasminogen activator system may be involved in the pathogenesis of pain. Urokinase-type plasminogen activator receptor mRNA was found in small and large DRG neurons (Hayden and Seeds, 1996; Siconolfi and Seeds, 2001). Sciatic nerve crush leads to elevated expression of uPAR, tissue-type plasminogen activator (tPA), and urokinase plasminogen activator (uPA) in DRG neurons (Siconolfi and Seeds, 2001). In the future, it will be important to determine the underlying mechanisms for the release of suPAR into circulation under acute or subacute pain conditions and to investigate the role of the plasminogen activator system in acute pain or subacute pain development.

### Function of TSP-2 in health and disease

Thrombospondin-2, a matricellular glycoprotein of the thrombospondin family, regulates multiple biological functions, including proliferation, angiogenesis, cell adhesion, and extracellular matrix modeling (Bornstein et al., 2000; Adams and Lawler, 2011). High TSP-2 levels in human sera and tissues have been reported in various patients (Neuschwander-Tetri et al., 2006; Farrow et al., 2009; Naumnik et al., 2015; Liu et al., 2018). Herein, we demonstrated a high serum TSP-2 level in patients with pain. Although the exact mechanisms linking serum TSP-2 and pain conditions are not well-understood, it has been found that TSP-4, another member of the thrombospondin family, contributes to spinal centralization by regulating the calcium channel Cavα2δ-1 in the spinal dorsal horn (Park et al., 2016). Therefore, investigation of TSP-2 expression and the local effects of TSP-2 in the pain pathway may provide clues on how TSP-2 is involved in pain pathogenesis.

We found significant changes in OPN, TSP-2, sPECAM-1, sErbB2, suPAR, and sIL-6Rα levels in the serum of patients with pain. Moreover, the serum OPN concentration may indicate pain severity. The results from this study provide new valuable insights into our understanding of angiogenic factors that may contribute to mechanistic biomarkers of pain to reveal the pathophysiological mechanisms of acute and subacute pain.

## Summary

The present study showed significant changes in OPN, TSP-2, sPECAM-1, sErbB2, suPAR, and sIL-6Rα levels in the serum of patients with pain, especially with PHN. Moreover, the serum OPN concentration may indicate pain severity. Based on our findings, we hypothesized that elevated levels of circulating angiogenic factors contribute to the development of pain pathogenesis. Further studies should focus on their local effects and functions in the pain pathway, such as in the dorsal root ganglion and spinal cord, to determine their contribution to pain pathogenesis. To date, the expression of genes encoding these altered angiogenic factors has been found to be increased in the injured DRG in an experimental neuropathic pain model based on RNA-sequencing analysis (Wu et al., 2016). However, it needs to be noted that the correlation of circulating angiogenic factors and their local expression is not always consistent. For example, investigation of EGFR and AXL in DRG sensory neurons showed that they are involved in the peripheral mechanism of neuropathic pain although we didn’t observe a significant change in sEGFR and sAXL in the sera of patients with pain (Martin et al., 2017; Wang et al., 2019). Therefore, serum indices unquestionably enlighten some ideas on pain pathogenesis, but further experimental animal studies need to be performed to explore precise mechanisms.

## Study limitations

A limitation of our study was the study group selection. Healthy controls were chosen from among age-matched elderly individuals who may have various other diseases, which may have affected serum angiogenic factor concentrations. Another limitation of this study was the inadequate number of patients, especially those with LBP and TN. We recruited only one patient with TN; therefore, the data could not be statistically analyzed. Further large-scale studies are required to define the role of these markers in patients with pain.

## Data availability statement

The raw data supporting the conclusions of this article will be made available by the authors, without undue reservation.

## Ethics statement

The studies involving human participants were reviewed and approved by the Ethical Committee of Tianjin Union Medical Center. The patients/participants provided their written informed consent to participate in this study.

## Author contributions

XY and JZ: conceptualization. HW and YW: methodology. HW and ML: formal analysis. XY and CY: writing—original draft preparation. JZ and ZL: writing—review and editing. CY: visualization. XY: supervision. JZ and XY: project administration and funding acquisition. All authors have read and agreed to the published version of the manuscript.

## Conflict of interest

The authors declare that the research was conducted in the absence of any commercial or financial relationships that could be construed as a potential conflict of interest.

## Publisher’s note

All claims expressed in this article are solely those of the authors and do not necessarily represent those of their affiliated organizations, or those of the publisher, the editors and the reviewers. Any product that may be evaluated in this article, or claim that may be made by its manufacturer, is not guaranteed or endorsed by the publisher.
